# Supplementary material for: Redefining the Prognostic Value of High-Sensitivity Troponin in COVID-19 Patients: The Importance of Concomitant Coronary Artery Disease
Source: J Clin Med. 2020 Oct 12;9(10):3263. doi: 10.3390/jcm9103263 (PMC7601151; doi:10.3390/jcm9103263)
Supplement: Supplementary file 1 [file jcm-09-03263-s001.pdf]

SUPPLEMENTARY MATERIALS

Tables S1-S2A-S2B
Multivariable logistic regression analysis showing independent overall and by group predictors of in-hospital mortality (see also Figure 1 – panel A and B).

Table 1. Overall mortality predictors in multivariate logistical regression.

	OR	IQR	*p*
**PaO2/FiO2 < 300 mmHg**	8.0	4.5-14.2	<0.001
**Heart failure**	4.7	2.0-10.8	<0.001
**Age > 65 years**	3.2	1.7-6.2	0.001
**Male gender**	2.6	1.4-4.8	0.002
**Myocardial injury**	2.5	1.3-4.5	0.003
**CCS**	2.3	1.1-5.1	0.03
**Diabetes**	2.1	1.2-4.0	0.015
**Obesity**	1.6	0.6-4.1	0.312
**COPD**	1.5	0.6-3.9	0.366
**CKD**	1.1	0.5-2.4	0.785
**Smoke**	0.8	0.4-1.8	0.603
**Hypertension**	0.8	0.4-1.4	0.374

Table S2: stratification of in-hospital mortality between CCS and non-CCS patients;.

**Table S2A: Mortality Predictors in CCS patients**			
	**OR**	**IQR**	*p*
**Age > 65 years**	4.2	0.8-23.3	0.100
**Male gender**	5.4	1.5-19.5	0.011
**Hypertension**	1.8	0.4-7.4	0.426
**PaO2/FiO2 < 300 mmHg**	10.4	3.5-31.0	<0.001
**Heart failure**	2.9	0.9-9.2	0.064
**CKD**	1.1	0.3-3.9	0.857
**COPD**	1.5	0.3-7.9	0.629
**Myocardial injury**	0.6	0.2-1.9	0.409
**Obesity**	3.3	0.5-20.0	0.197
**Diabetes**	1.5	0.5-4.7	0.497
**No previous revascularization**	2.4	0.6-9.1	0.194
**Table S2B: Mortality Predictors in non-CCS patients**			
	**OR**	**IQR**	*p*
**Age > 65 years**	2.8	1.3-6.2	0.01
**Male gender**	2.0	0.9-4.3	0.078
**Hypertension**	0.5	0.2-1.1	0.081
**PaO2/FiO2 < 300 mmHg**	6.0	2.9-12.3	<0.001
**Heart failure**	16.3	3.4-77.3	<0.001
**CKD**	1.1	0.4-3.2	0.879
**COPD**	1.2	0.3-3.9	0.809
**Myocardial injury**	5.0	2.4-10.6	<0.001
**Obesity**	1.5	0.4-5.2	0.543
**Diabetes**	2.7	1.2-6.1	0.019
**No previous revascularization**	NA	NA	NA

Abbreviations: CCS: chronic coronary syndromes; CKD: chronic kidney disease; COPD: chronic obstructive pulmonary disease; FiO2: fraction of inspired oxygen; IQR: interquartile range; NA: not applicable; OR: odds ratio; paO2: partial pressure of oxygen in arterial blood.

Tables S3-S4A-S4B
Multivariable logistic regression analysis showing independent overall and by group predictors of myocardial injury (see also Figure 1 – panel C and D).

Table 3. Overall Myocardial Injury Predictors in Myocardial Injury Predictors.

	OR	IQR	p
**Diabetes**	3.42	1.85 – 6.36	< 0.001
**Age > 65 years**	3.28	1.68 – 6.40	0.001
**Heart failure**	3.02	1.21 – 7.55	0.018
**Hypertension**	2.10	1.18 – 3.75	0.012
**CCS**	0.69	0.30 – 1.55	0.371
**Smoke**	1.06	0.48 – 2.34	0.880
**PaO2/FiO2**	1.00	0.99 – 1.00	0.090
**Male gender**	0.96	0.55 – 1.68	0.896
**Hb**	0.87	0.76 – 0.99	0.038
**COPD**	1.01	0.40 – 2.48	0.998
**CKD**	0.48	0.20 – 1.13	0.093
**Obesity**	0.62	0.22 – 1.74	0.362

Table 4. stratification of myocardial injury between CCS and non-CCS patients.

**Table S4A: Myocardial Injury Predictors in CCS patients**			
	**OR**	**IQR**	p
**Diabetes**	3.41	1.02 – 11.38	0.046
**Age > 65 years**	0.71	0.12 – 4.11	0.706
**Heart failure**	2.66	0.67 – 10.45	0.160
**Smoke**	1.87	0.52 – 6.73	0.339
**Hypertension**	1.62	0.38 – 6.84	0.515
**Male gender**	0.57	0.15 – 2.01	0.370
**Hemoglobin**	0.84	0.61 – 1.16	0.288
**PaO2/FiO2**	1.01	0.99 – 1.00	0.171
**COPD**	1.24	0.26 – 5.79	0.785
**CKD**	0.65	0.14 – 2.92	0.572
**Obesity**	0.50	0.05 – 4.55	0.541
**No previous revascularization**	2.48	0.60 – 10.20	0.209
**Table S4B: Myocardial Injury Predictors in non-CCS patients**			
	**OR**	**IQR**	p
**Diabetes**	3.06	1.35 – 6.82	0.007
**Age > 65 years**	3.92	1.84 – 8.40	< 0.001
**Heart failure**	5.23	0.98 – 28.04	0.052
**Smoke**	1.02	0.33 – 3.14	0.965
**Hypertension**	1.85	0.93 – 3.67	0.078
**Male gender**	1.38	0.71 – 2.70	0.347
**Hemoglobin**	0.85	0.73 – 0.98	0.033

**PaO2/FiO2**	0.99	0.99 – 0.99	0.004
**COPD**	0.88	0.25 – 3.04	0.835
**CKD**	0.58	0.18 – 1.81	0.347
**Obesity**	0.87	0.12 – 14.4	0.819

Abbreviations: CCS: chronic coronary syndromes; CKD: chronic kidney disease; COPD: chronic obstructive pulmonary disease; FiO2: fraction of inspired oxygen; IQR: interquartile range; OR: odds ratio; paO2: partial pressure of oxygen in arterial blood.
